# Mild hypothermia therapy improves neurologic outcomes in patients with acute stroke

**Published:** 2012-11

**Authors:** Rostam Jalali, Behnam Khaledi Paveh, Maryam Hayati

**Affiliations:** ^*a*^Kermanshah University of Medical Sciences, Kermanshah, Iran.; ^*b*^Imam Reza Hospital, Kermanshah University of Medical Sciences, Kermanshah, Iran.

## Abstract

**Background:**

In acute stroke patients, mild and moderate hypothermia with a body temperature (T core) target of 32 °C to 34 °C has been tested with some promising results. Clinicians have been increasingly using therapeutic hypothermia to prevent or control various types of neurological injury. In this review study we evaluate the current clinical evidence regarding the use of therapeutic hypothermia.

**Methods:**

A review of the literature was conducted usining PubMed database.

**Results:**

Various cooling techniques and devices have been developed and assessed for the treatment of acute stroke patients. The efficacy of these new methods and devices has been demonstrated in many preliminary studies.

**Conclusions:**

Regarding the findings of this survey, hypothermia can cause unwanted side-effects, thus further research is needed to conduct quantified risk assessments as well as therapeutic benefits assessment of hypothermia.

**Keywords:**

Acute ischemic stroke, Mild hypothermia, Neurological outcome


					Volume 4, Suppl. 1 Nov 2012
Publisher: Kermanshah University of Medical Sciences
URL: http://www.jivresearch.org
Copyright:
Congress of Iranian Neurosurgeons, 14-16 November 2012, Kermanshah, Iran
Paper No. 57
Mild hypothermia therapy improves neurologic outcomes in
patients with acute stroke
Rostam Jalali a,*
, Behnam Khaledi Paveh a
, Maryam Hayati b
a
Kermanshah University of Medical Sciences, Kermanshah, Iran.
b
Imam Reza Hospital, Kermanshah University of Medical Sciences, Kermanshah, Iran.
Abstract:
Background: In acute stroke patients, mild and moderate hypothermia with a body temperature (T core) target of
32 C to 34 C has been tested with some promising results. Clinicians have been increasingly using therapeutic
hypothermia to prevent or control various types of neurological injury. In this review study we evaluate the current
clinical evidence regarding the use of therapeutic hypothermia.
Methods: A review of the literature was conducted using PubMed database.
Results: Various cooling techniques and devices have been developed and assessed for the treatment of acute
stroke patients. The efficacy of these new methods and devices has been demonstrated in many preliminary studies.
Conclusions: Regarding the findings of this survey, hypothermia can cause unwanted side-effects, thus further
research is needed to conduct quantified risk assessments as well as therapeutic benefits assessment of hypothermia.
Keywords:
Acute ischemic stroke, Mild hypothermia, Neurological outcome
* Corresponding Author at:
Rostam Jalali: PhD of Nursing, Kermanshah University of Medical Sciences, Kermanshah, Iran. Tel: 09181324821,
Email: ks_jalali@yahoo.com, (Jalali R.).

				

